# Geographic Differences in Cannabis Conversations on Twitter: Infodemiology Study

**DOI:** 10.2196/18540

**Published:** 2020-10-05

**Authors:** Jenna van Draanen, HaoDong Tao, Saksham Gupta, Sam Liu

**Affiliations:** 1 Department of Sociology University of British Columbia Vancouver, BC Canada; 2 Department of Computer Science University of Victoria Victoria, BC Canada; 3 School of Exercise Science, Physical & Health Education University of Victoria Victoria, BC Canada

**Keywords:** marijuana, social media, sentiment, cannabis, Twitter

## Abstract

**Background:**

Infodemiology is an emerging field of research that utilizes user-generated health-related content, such as that found in social media, to help improve public health. Twitter has become an important venue for studying emerging patterns in health issues such as substance use because it can reflect trends in real-time and display messages generated directly by users, giving a uniquely personal voice to analyses. Over the past year, several states in the United States have passed legislation to legalize adult recreational use of cannabis and the federal government in Canada has done the same. There are few studies that examine the sentiment and content of tweets about cannabis since the recent legislative changes regarding cannabis have occurred in North America.

**Objective:**

To examine differences in the sentiment and content of cannabis-related tweets by state cannabis laws, and to examine differences in sentiment between the United States and Canada between 2017 and 2019.

**Methods:**

In total, 1,200,127 cannabis-related tweets were collected from January 1, 2017, to June 17, 2019, using the Twitter application programming interface. Tweets then were grouped geographically based on cannabis legal status (legal for adult recreational use, legal for medical use, and no legal use) in the locations from which the tweets came. Sentiment scoring for the tweets was done with VADER (Valence Aware Dictionary and sEntiment Reasoner), and differences in sentiment for states with different cannabis laws were tested using Tukey adjusted two-sided pairwise comparisons. Topic analysis to determine the content of tweets was done using latent Dirichlet allocation in Python, using a Java implementation, LdaMallet, with Gensim wrapper.

**Results:**

Significant differences were seen in tweet sentiment between US states with different cannabis laws (*P*=.001 for negative sentiment tweets in fully illegal compared to legal for adult recreational use states), as well as between the United States and Canada (*P*=.003 for positive sentiment and *P*=.001 for negative sentiment). In both cases, restrictive state policy environments (eg, those where cannabis use is fully illegal, or legal for medical use only) were associated with more negative tweet sentiment than less restrictive policy environments (eg, where cannabis is legal for adult recreational use). Six key topics were found in recent US tweet contents: fun and recreation (keywords, eg, love, life, high); daily life (today, start, live); transactions (buy, sell, money); places of use (room, car, house); medical use and cannabis industry (business, industry, company); and legalization (legalize, police, tax). The keywords representing content of tweets also differed between the United States and Canada.

**Conclusions:**

Knowledge about how cannabis is being discussed online, and geographic differences that exist in these conversations may help to inform public health planning and prevention efforts. Public health education about how to use cannabis in ways that promote safety and minimize harms may be especially important in places where cannabis is legal for adult recreational and medical use.

## Introduction

Social media platforms represent an important venue for studying emerging patterns in public health issues such as substance use because they can reflect trends in real time and display messages generated directly by users, giving a uniquely personal voice to analyses. This method of using social data to improve our understanding of public health is known as Infodemiology [1]. Twitter is a widely used social networking platform where users share messages in 280-character tweets (or 140-character tweets before October 2017), offering a rich venue for investigating public sentiment.

One of the main approaches to analyze unstructured text data from Twitter is sentiment of the tweets [2,3]. Sentiment analysis can determine whether an individual’s attitude or perception toward a topic is positive, negative, or neutral. A second popular method of analyzing tweets is topic modeling, which refers to a technique that discovers the hidden semantic structure in a text corpus. Topic modeling can be particularly helpful in providing insights into the different themes present in the texts [4,5]. By applying these methods, emerging research has used Twitter data for public health surveillance including monitoring symptoms of depression [6], psychological distress [7], influenza transmission [8], lifestyle behaviors [9], and substance use trends [10,11].

Twitter data can be especially useful in highlighting emergent areas of concern in substance use research that are not detectable from other sources, and past studies have uncovered critical issues, for example, related to the use of Adderall by adolescents [10], procannabis content being disproportionately created and consumed by young minorities [12,13], and geographic variation in discussions about cannabis concentrate (dabs) [14]. Prior research in this area has begun to characterize the sentiment of cannabis-related tweets both manually [15,16] and using advanced information processing techniques [17]. Sentiment toward cannabis online has been characterized as dominantly procannabis, and this is especially the case in personal communications, which make up the strong majority of tweets about cannabis [17]. As the policy landscape related to cannabis is rapidly changing, so too may attitudes toward cannabis online, but this is currently unknown.

Several states in the United States have passed legislation to legalize adult recreational use of cannabis (eg, Colorado, Washington, California) and the federal government in Canada has done the same [18,19]. These legislative changes may be both reflecting and impacting public sentiment about cannabis use. For example, states that have less restrictive policies about cannabis use have historically been found to have a higher volume of cannabis-related tweets [14,20] and the volume of procannabis tweets has previously been found to increase postlegalization. Further, tweets from states with less restrictive policies are more positive and vary according to the local social and demographic trends [21]. Using 2016 data, Daniulaityte and colleagues [20] compared sentiment toward cannabis in tweets by legality of cannabis at the state level and found statistically significant differences in the ratio of positive to negative tweets. States with recreational laws had a mean ratio of 4.64 (favoring procannabis tweets) and states where all consumption of cannabis was illegal had a ratio of 4.19 [20]. Similarly, positive sentiment in tweets about rosin (a cannabis concentrate) is up to 16 times higher in states allowing adult recreational use of cannabis compared with states where cannabis is illegal [22]. In the 3 years since the last sentiment analysis, the policy environment surrounding cannabis has changed dramatically and it is possible that users of Twitter have changed the way they talk about cannabis online as well; however, this is currently unknown.

Previous content analysis has indicated that themes among procannabis adolescent Twitter users included intent to use or craving; frequent, heavy, or regular use; health benefits and prolegalization; sex/romance; attractiveness or friendships; and current use [15]. A study of influential tweets (ie, tweets with a high number of re-tweets) about both alcohol and cannabis found that common themes included using marijuana or alcohol with friends, sex/romance, and tobacco or other drugs [11]. However, these content analyses had samples restricted to influential Twitter users and adolescents [12,13,15,23]. Another recent study of tweets from a more general sample captured 12 topics in cannabis-related tweets and also found polysubstance use to be a recurring topic, in addition to topics such as using cannabis, health and medical, cannabis industry, and legality (among others) [24].

Given that the way both governments and the general public are interacting with cannabis is rapidly changing in North America, and that Twitter data have been shown to illustrate trends in real time, it is timely to conduct an updated analysis of the sentiment and content of tweets about cannabis. The objectives of this study are, therefore, to examine differences in the sentiment and content of cannabis-related tweets by state cannabis laws and between the United States and Canada. To our knowledge, this is the first analysis that includes Canadian data and the first analysis to be conducted with data up to 2019.

## Methods

### Data Collection and Cleaning

Tweets were collected from January 1, 2017, to June 17, 2019, using the Twitter Realtime Filter application programming interface (API) and a standard access token [25]. Tweets were streamed using a location box of the following longitude and latitude (–162.354635, 18.756125, –53.755999, 73.893030). We captured 576 million tweets. Twitter API used in this study captures a random sample of about 1% of all tweets in Canada and the United States in the designated period. University institutional review board approval was not required as the data set was limited to publicly available tweets. These tweets were stored in “.csv” files and resampled using custom Python script with Pandas library. Each tweet has 3 nonmandatory attributes to determine location by state or province: *tweetLocation* (an identifiable address indicating the rough location from where the tweet was sent), and 2 geo coordinate attributes, namely, *Longitude* and *Latitude.* Because our research questions are fundamentally concerned with place-based legislation changes, we retained only tweets with location data in our data set. Tweets that that were linked with location attributes were labeled using a custom-built dictionary that maps the location strings to the 2-letter state or province abbreviations. Tweets with geocoordinates only were labeled with reverse geocode lookup using Google Maps API.

In the US data set, 50 states and 1 federal district (District of Columbia) were grouped based on cannabis legal status [26]. These 3 groups include (1) Fully illegal states for both adult recreational and medical use (n=12; Alaska, Indiana, Iowa, Kansas, Kentucky, Missouri, North Carolina, Nevada, South Carolina, South Dakota, Tennessee, Wyoming); (2) legal for medical use only (n=30; Arkansas, Arizona, California [up to January 1, 2018], Connecticut, Delaware, Florida, Georgia, Hawaii, Idaho, Illinois, Louisiana, Maryland, Michigan [up to December 5, 2018], Minnesota, Mississippi, Montana, Nebraska, New Hampshire, New Jersey, New Mexico, New York, Ohio, Oklahoma, Pennsylvania, Rhode Island, Texas, Utah, Virginia, Vermont [up to June 30, 2018], West Virginia); and (3) legal for both adult recreational and medical use (n=11; Alabama, California [starting January 1, 2018], Colorado, District of Columbia, Massachusetts, Maine, Michigan [starting December 6, 2018], Vermont [starting July 1, 2018], North Dakota, Oregon, Washington). Three US states (Michigan, California, and Vermont) changed their cannabis regulatory policy during the data collection period and thus tweets from these 3 states were grouped into different categories depending on whether they were posted prelegalization or postlegalization.

Because Canada had no legal differences across provinces in cannabis law and only minor regulatory differences, variation in tweets across Canada by provincial legal status was not examined. Nationally, cannabis became legal for medical use in Canada in 2001 (with the Marihuana for Medical Purposes Regulations) and became legal for adult recreational consumption on October 17, 2018 with the passage of Bill C-45: Cannabis Act in June 2018 [18].

### Classifying Marijuana Related Tweets

Tweets were classified as being cannabis-related using a set of keywords based on the method used by others in the field [27]. We translated the following search queries and conditions into regular expressions: “Weed,” “marijuana,” “cannabis,” “smoke AND (pot OR joint OR blunt OR mary jane),” “need AND (pot OR joint OR blunt),” “want AND (pot OR a blunt),” and “want AND a joint.” Furthermore, for queries with 2 search terms, the terms were required to be within 3 words of each other, as done in related studies [27]. In total, 1,200,127 tweets were classified as cannabis related: 1,149,137 from the United States and 50,990 from Canada.

### Classifying Sentiment

Sentiment scoring for the tweets was done with VADER (Valence Aware Dictionary and sEntiment Reasoner) Sentiment, a lexicon and rule-based sentiment analysis tool [3]. VADER analysis uses a predefined dictionary that maps on different words and lexicon features, acronyms, and slang to determine the positive or negative sentiment of a tweet [28]. Sentiment valence score increases with words with more positive sentiment (eg, happy, nice, good). The tweets were parsed through the VADER sentiment analysis to check for the words, emoticons, and slang that are present in the lexicon. VADER also gave importance to capital letters and exclamation marks. VADER calculated the proportion of positive, neutral, and negative sentiment scores for a tweet. A compound score was then calculated by summing the sentiment scores and normalizing the result. Sentiment scores can range between –1 (negative sentiment) and 1 (positive sentiment). Scores between –0.05 and 0.05 were considered neutral. Normalization was performed using the following formula: z/√(z^2^+r), where z is the value created after adding the valence scores of a tweet, and r is a normalization constant which is taken as 15 by default. The code for our analysis was made available online [29]. Classifying tweets associated with substance use using automated methods can be particularly difficult due to the use of slang and implied meanings [17]. A tweet’s sentiment does not necessarily reflect the Twitter user’s stance on cannabis usage; it instead reflects the emotionality in their words used to communicate about cannabis, thus positive sentiment is not the same as a procannabis opinion, and likewise, negative sentiment is not analogous with an anticannabis perspective. The null hypotheses of no difference in proportions between groups of states defined by legal status and between the United States and Canada were tested with two-sided pairwise comparisons using Stata version 14.0 (StataCorp). Multiple comparisons were adjusted for using the Tukey method [30].

### Topic Modeling

Topic analysis to determine the content of tweets was done using latent Dirichlet allocation (LDA) in Python, using a Java implementation, LdaMallet, with Gensim wrapper [31]. LDA is an unsupervised probabilistic model which generates mixtures of topics from a corpus of text. A topic is a probability distribution over every word found in the corpus. LDA works by looking at the word co-occurrences within the corpus of text, assuming that words that occur in the same corpus of text are more likely to be on the same topic than words that are not [32]. The UMass coherence matrices were used to measure topic coherence. A previous study has shown that this method achieved reasonable results when comparing the scores obtained by this measure with human scoring on a corpus of 300,000 health journal abstracts [4]. The model with the highest coherence score was chosen for analysis. We then used human judgment to validate the topics identified. The use of a human’s perception has been previously used along with statistical methods to evaluate topic models using Twitter [33,34]. Specifically, we performed word and topic intrusion tasks. Word intrusion allows the analyst to measure how semantically *cohesive* the topics inferred by a model are and tests whether topics correspond to natural groupings for humans. Topic intrusion enabled the human subject to evaluate how well a topic model in a document as a mixture of topics agrees with human associations of topics with a document [33]. LDA analyses were conducted separately for the US and Canada data sets. It is possible that a single tweet can contain multiple topics.

## Results

**Figure 1:**
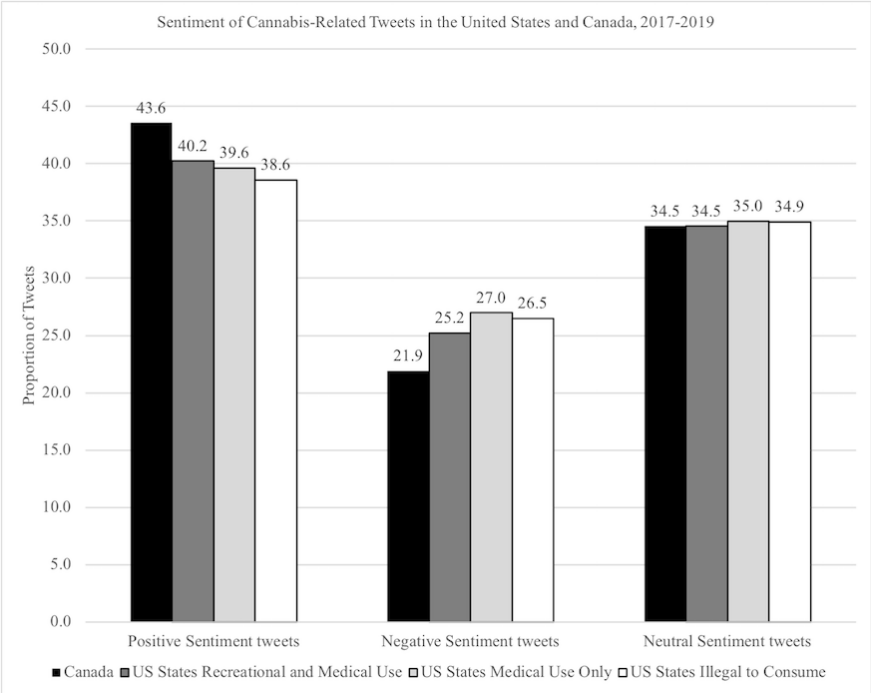
Sentiment of Cannabis-Related Tweets in the United States and Canada, 2017-2019.

Overall, most tweets were of positive sentiment (39.39% [472,746/1,200,127]) and proportions of neutral (34.50% [414,006/1,200,127]) and negative sentiment tweets were lower (26.11% [313,357/1,200,127]) in all data sets. Examples of tweets that were recorded in the positive sentiment category include “I love being from the Westside. Cooler weather and better Weed,” and “Weed is just so great. And food is so great. And music is great. You’re all great. Everything’s great lol.” While negative sentiment tweets included, for example, “I hate the smell of weed. Hate it. Hate it. Hate it. Hate it. Hate it. Hate it. Hate it. Hate it. Hate it. Hate it. Hate it. Hate it. Ha...” and “The marijuana prohibition has created more crime than alcohol prohibition did and is a bad idea.” Neutral sentiment tweets included, for example, “who would wanna smoke more weed to get less out of it...” and “Smoke this blunt then I'm sleep.” Figure 1 depicts the proportion of positive, negative, and neutral sentiment tweets in Canada and the United States.

As can be seen in Table 1, the Canadian data set had a higher proportion of positive sentiment tweets than the United States data set overall (43.56% [22,210/37,050,924] vs 39.62% [325,699/587,058,027]; *P*=.003) and a lower proportion of negative tweets (21.90% [11,169/37,050,924] vs 26.99% [221,876/587,058,027]; *P*=.001). Differences are apparent in the proportion of negative sentiment tweets from states with adult recreational and medical, medical use only, and no legal cannabis use policies (Table 1). The proportion of negative tweets was lower in states where adult recreational use was legal (25.22% [56,601/143,784,107]) than states where cannabis is illegal to consume (26.50% [23,711/98,092,868]; *P*<.001). States with more restrictive laws regarding cannabis use also had a smaller proportion of tweets that were cannabis related. See Table 1 for a summary of the sentiment analysis. See Multimedia Appendix 1 for full data analysis.

**Table 1:** Sentiment of cannabis-related tweets in the United States and Canada, January 2017-2019.

Tweet sentiment		Canada (N=37,050,924), n (%)	Pairwise comparison, P-values	US States Recreational and medical Use (1) (N=143,784,107), n (%)	US States Medical Use only (2) (N=587,058,027), n (%)	US States Illegal to consume (3) (N=98,092,868), n (%)	Pairwise comparison, *P*-values (Tukey adjusted)
**Cannabis-related tweets**		50,990 (0.14)	Canada vs United States: .375	224,462 (0.16)	821,970 (0.14)	89,482 (0.09)	1 vs 2: <.001^a^ 2 vs 3: <.001^a^ 1 vs 3: <.001^a^
	Positive sentiment tweets	22,210 (43.56)	Canada vs United States: .003^a^	90,331 (40.24)	325,699 (39.62)	34,524 (38.58)	1 vs 2: .630 2 vs 3: .367 1 vs 3: .151
	Negative sentiment tweets	11,169 (21.90)	Canada vs United States: .001^a^	56,601 (25.22)	221,876 (26.99)	23,711 (26.50)	1 vs 2: .596 2 vs 3: <.001^a^ 1 vs 3: <.001^a^
	Neutral sentiment tweets	17,611 (34.54)	Canada vs United States: .887	77,530 (34.54)	287,618 (34.99)	31,247 (34.92)	1 vs 2: >.99 2 vs 3: .218 1 vs 3: .320

^a^Statistical significance at the .05 level of significance.

Content analysis was done separately for tweets from the United States and Canada, to allow for detection of country-specific themes. In the US data set, Topic 1 contained tweets about the author having fun and getting high. Example tweets include “I’m glad we’ve gotten closer over the last year, I love smoking pot and talking shit with you in between shows” and “I love just smoking a blunt with nick and we can talk about anything.” Topic 2 was similar but distinct and contained tweets about living life and smoking cannabis and frequently included words such as smoke, people, make, today, live, start, day, and happy. Example tweets from Topic 2 include “Keep your head high and your joint higher” and “I live in Colorado and I'm artsy so I have like...an ounce of weed in my bag.” Topic 3 contained tweets related to transactions and also contained a racial slur as a keyword that came up frequently in posts that fell into this category. Example tweets include “Need to go buy bud to knock the fuck out,” and “You know the struggle when you can’t decide whether to spend money on weed or make up.” Topic 4 related to the places that people use cannabis and the associated scent. Example tweets include “My car doesn’t smell like pot anymore,” and “That moment when your dad follows you into your room and smells something funky but does realize it's just weed.” Topic 5 contained tweets related to medical use and the cannabis industry with keywords such as medical, grow, business, industry, dispensary, company, health, and medicine. Example tweets include “What are your thoughts on the medical marijuana business (or recreational)? Worth investing?” and “If you’re looking for work in a growth industry, marijuana is booming.” Lastly, Topic 6 was about legalization and criminalization. Example tweets include “As CA business we fought to end civil rights disaster of arresting marijuana users. Will your medical cannabis policy be guided by science or disproven statements?” and “For so long government wanted to criminalize marijuana but now they're just trying to make sure they're on the winnin if end of legalization.” See Table 2 for a list of the topic areas in the US data set and their frequency by state legal grouping.

Content analysis was also performed on the Canadian data set and similar topics were found with some slight differences (Table 3). Notably, themes related to everyday life and fun/recreation were present in a single topic area in the Canadian data set. Example tweets include “The summer will be well deserved. I plan on being intoxicated by alcohol or cannabis 90% of the time” and “As I wake n bake I'd like to acknowledge Mary Marijuana who has been amazing to me over many decades.” Topics related to medical use and the cannabis industry were 2 distinct themes. Example medical use tweets include “Fibromyalgia Targeted in AP-CBD/THC Therapy Now Undergoing Phase 1 Clinical Trial” and “Canada’s largest pharmacy chain says its own employees would be covered for medical marijuana prescriptions.” Example cannabis industry tweets include “Canadian cannabis industry raises $700M in six months” and “Recalls force medical marijuana industry to step up quality testing threatening the pot industry to enforce federal laws to ban growing and selling marijuana.” The Canadian tweets contained frequent references to geographic places including cities and provinces. Finally, the Canadian data set contained minimal racial slurs in the topic about personal transactions, even after we tested specifically for the presence of this clustering of words.

**Table 2:** Topics in cannabis-related tweets by state legal category groupings, United States, January 2017-2019.

Topics	Theme keywords	Proportion of tweets in Theme^a^				
		US States Recreational and medical use % (N=224,462)^b^	US States Medical use only % (N=821,970)^b^	US States Illegal to consume % (N=89,482)^b^	Overall % (N=1,135,914)^b^	
1. Fun and recreation	Smoke, weed, blunt, fuck, lol people, love, life, day, high	26.25	31.24	33.20	30.21	
2. Daily life	Weed, smoke, people, make, today, live, pot, start, day, happy	24.07	24.21	23.64	24.00	
3. Transactions	Weed, buy, money, sell, shop, spend, store, make, deal, [*racial slur*]	11.52	11.77	13.00	12.13	
4. Places of use	Weed, smell, car, smoke, room, mom, walk, house, marijuana, straight	11.81	12.31	12.53	12.23	
5. Medical use/Cannabis industry	Marijuana, cannabis, medical, grow, business, industry, dispensary, company, health, medicine	16.01	9.90	7.31	10.99	
6. Legalization	Marijuana, legal, legalize, state, cannabis, illegal, police, tax, legalization, arrest	10.34	10.56	10.32	10.44	

^a^There are no n values supplied for the content analysis as tweets may have multiple topics.

^b^The N value corresponds to total cannabis-related tweets.

**Table 3:** Topics found in content analysis for cannabis-related tweets by state legal category groupings, Canada, January 2017-2019 (N=50,990).

Topics	Theme keywords	Proportion of tweets in theme, %^a^
1. Everyday life, Fun, Recreational use	Weed, smoke, people, day, today, fuck, high, love, alcohol, start	12.60
2. Cannabis industry	Cannabis, grow, marijuana, industry, market, company, sales, business, book, support	27.33
3. Transactions	Weed, sell, buy, marijuana, store, cannabis, Canada, order, money, Ontario	9.06
4. Places of use	Weed, smell, marijuana, smoke, time, make, Toronto, bad, walk, call	24.84
5. Medical use	Cannabis, medical, marijuana, health, dispensary, tax, dispensaries, medicine, pharma	6.68
6. Legalization	Marijuana, cannabis, legal, legalize, legalization, Canada, cdnpoli, police, justintrudeau, government	19.49

^a^There are no n values supplied for the content analysis as tweets may have multiple topics.

## Discussion

### Summary of Findings

Our study presents novel findings from a recent sentiment and content analysis of Twitter data, and is the first study to compare trends in online discussions about cannabis in Canada and the United States. Our analysis of cannabis-related tweets from January 2017 to 2019 found differences in the sentiment and content of tweets from states with adult recreational, medical, and no legal cannabis use policies, and Canada. States with more restrictive laws regarding cannabis use had both a smaller proportion of tweets that were cannabis-related and a higher proportion of tweets that had a negative sentiment than those with less restrictive laws. The US tweets overall contained a higher proportion of negative tweets and a lower proportion of positive tweets than tweets from Canada. This may be indicative of changes in public opinion, becoming more positive after legalization of cannabis, or it may simply be that people are more comfortable sharing positive opinions and emotions in public, online forums when there are no potential legal ramifications for their actions.

The content analysis done on the US data set revealed some similar themes to previous content analyses such as current use and legalization, transactions, medical use, and the cannabis industry [13,24]; however, our analysis also presented new themes not uncovered in previous content analyses including the topics about the scent of cannabis and places that people use, and medical use and the cannabis industry. Our analysis did not detect several of the themes that other authors did, such as romance, tobacco, and friendship [15] or processed product use, cannabidiol and hemp use, and polysubstance use [24]. This could be because previous content analyses were conducted primarily with influential Twitter users, with adolescent users, and not with all available tweets [12,13,15,23] or because of a more expansive time frame and fewer geographic restrictions than other studies [24].

In the United States, tweets in Topic 5 (medical use and the cannabis industry) appear to be more common in states that have legalized cannabis for adult recreational use, possibly because these states have regulated environments for purchase and consumption of cannabis, and Twitter users in these places have more interaction with the cannabis industry. Similarly, Topic 3 (personal transactions) was more frequently found coming from Twitter users in states where all cannabis use was illegal, likely stemming from the need to buy and sell cannabis in an underground market, consisting of individual transactions.

In the Canadian data set, similar topics were found with some slight differences. Topics related to medical use and the cannabis industry were 2 distinct themes in Canada, likely because adult recreational use was legalized in the study period and the regulatory environment separated medical and adult recreational use (both being or becoming legal, but sold and regulated in different ways).

Legalization was also discussed differently in the 2 data sets. The Canadian discussions of legalization were more politicized with terms such as *justintrudeau* and *canpoli* coming up frequently, whereas the US legalization theme included the words *police* and *arrest.* These differences may reflect cannabis legalization being a national political issue in Canada, as it was tied to party platforms in the 2015 federal election cycle and became a major campaign promise of the Liberal government. These differences may also reflect the persistent enforcement of low-level drug offences in the United States and the relatively high levels of incarceration associated with cannabis in the United States (making up 40%-50% of drug charges) [29].

Other differences between the themes within the Canadian and US data sets include the frequency of the presence of the word *cannabis* in the Canadian tweets. This is the term preferred by the Canadian government, and evidently has spread into personal communications. In the US data set, slang words for cannabis were more frequently present including *weed* and *pot* and *blunt*, possibly because of the absence of discussions that use more formal language in national policy spaces permeating into personal communications, or possibly because of differences in who is tweeting about cannabis. The word *marijuana* was frequently present in both data sets. In the Canadian data set, alcohol is included as a keyword in Topic 1, which is about recreational use, a term not found in the US clusters.

Finally, the Canadian data set contained fewer racial slurs in the topic about personal transactions, even after we tested specifically for the presence of this clustering of words. It may represent a troubling normalization of derogatory language toward African Americans in the United States, or it may be representing larger, structural issues in the racialization of drug enforcement [29,35] and perceptions of who is selling drugs. Although the proportion of White Americans who consume marijuana has repeatedly been reported to be either similar to or higher than the proportion of Black Americans, there are differences in racialized perceptions of use and racialized drug enforcement [29,35]. Alternately, it could represent an increase in online conversations about cannabis among minorities in the United States, which has also been documented in other studies [15].

### Limitations

Our analysis was limited to tweets with location data, English language content, and those from United States and Canada, limiting the generalizability of the findings. Further, studies have shown that there can be bias related to population demographics at the state and city level [36] as well as temporal and spatial factors at the individual level [37] that can affect the sentiment of tweets. Geotagged Twitter data are a subset of general Twitter data and may not accurately represent the wider population. For example, only 15% of adults who use the internet use Twitter with regularity, and those aged 18-29 as well as minorities tend to be more highly represented on Twitter than in the general population. There are higher proportions of both passive users (<50 tweets per year) and highly active users (>1000 tweets per year) than moderate users (50-1000 tweets per year) on Twitter [38]. Taken together, these limitations indicate that the data used in this study are from nonuniformly gathered statements from a nonrepresentative subset of the US and Canadian populations.

The analytic methods used to ascertain sentiment was based on the tone of words used in the tweets, and thus a tweet’s sentiment does not necessarily reflect the Twitter user’s stance on cannabis usage and does not translate to procannabis or anticannabis opinion. In stance detection, the analysis needs to determine favorability toward a given target of interest. The target of interest is often prechosen and may not be explicitly mentioned in the text and it may not be the target of opinion in the text [39]. Other scholars have performed sentiment analyses on cannabis-related tweets using different methods and restricting analysis to Twitter users having high Klout scores, finding closer to 65%-77% of tweets with a positive sentiment [13]. We expect the higher proportions of positive sentiment in the previously referenced paper are due to selecting users with a high Klout score, who have more positive sentiment overall than lower Klout score users, as well as their use of a custom classification method designed to capture the intent of the tweet rather than just the sentiment [13]. We manually classified 350 tweets ourselves based on emotional tone and compared these with VADER Sentiment and found that VADER was nearly 85% accurate with only 51 misclassified out of 350 (14.6%), giving us confidence in the sentiment results presented here.

Further methodological limitations include that we are not able to filter out bots from the data set, although we have removed duplicate tweets. Data from the 3 US states included that had cannabis law changes during the study period may not fully represent cannabis tweet sentiment in these states, and study results should not be generalized beyond the limited time frame in which data were collected. Finally, we were not able to retrieve tweets from accounts which were marked as private by the API.

### Public Health Implications

We document a notable difference in the sentiment of tweets whereby less restrictive policy environments appear to be associated with less negative sentiment in tweets and perceptible differences in Twitter content between the United States and Canada. The implications of this work extend beyond just online messaging. Cannabis is the most commonly used drug in both the United States and Canada, with between 13% and 18% of the general population reporting recent use [37,40]. Cannabis use and cannabis use disorder are significantly associated with higher levels of exposure to procannabis content on Twitter [12] and living in places with more liberal cannabis policies [37,41], suggesting that such exposures are consequential.

Other studies have documented the procannabis sentiment of much of the cannabis-related Tweets from the general public [12], and this taken in tandem with the documented lack of cannabis-related educational information on Twitter from health organizations [42] suggests that Twitter users (especially in states with less restrictive policy environments) may benefit from information regarding how to use cannabis in ways that minimize health-related harms. Some examples of educational messages designed to target adults in the general public and to help maximize safety and health when using cannabis include “start low and go slow” as well as “use cannabis in a safe and familiar environment with people you trust” and “if you are a new consumer, look for a product with less than 100 mg/g (10%) THC, with equal or higher levels of CBD,” etc [37].

Knowledge generated in this study about how cannabis is being discussed online, and geographic differences that exist in these conversations may help to inform public health planning and prevention efforts. For example, campaigns targeting specific geographic areas may be useful as cannabis laws become less restrictive in the United States. The content analysis conducted in this study highlighted some potential trends that deserve further investigation. Future research is needed on the racialized nature of cannabis conversations on Twitter, the potential role of social media in buying and selling cannabis, and emerging trends surrounding places of use (eg, use at home, use in public spaces).

## Appendix

Multimedia Appendix 1 Data analysis script and data file.

## Abbreviations

API: application programming interface
CBD: cannabidiol
LDA: latent Dirichlet allocation
THC: tetrahydrocannabinol
VADER: Valence Aware Dictionary and sEntiment Reasoner
