# Supplementary material for: Geographic Differences in Cannabis Conversations on Twitter: Infodemiology Study
Source: JMIR Public Health Surveill. 2020 Oct 5;6(4):e18540. doi: 10.2196/18540 (PMC7573699; doi:10.2196/18540)
Supplement: Multimedia Appendix 1 [file publichealth_v6i4e18540_app1.docx]

*Appendix.*

Access to data analysis code

<https://github.com/SakshamGupta55/NLP-on-tweets-from-social-media.git>

Access to Twitter data. Sharing of the twitter data is in accordance with Twitter policy^[[1]](#footnote-1)^.

<https://drive.google.com/drive/folders/1H3FngZBohfe8rxR6JX-Sn5suajCc9XQB?usp=sharing>

1. Twitter data sharing policy. Up to 50,000 public Tweets may be shared at a time. https://developer.twitter.com/en/developer-terms/policy1 [↑](#footnote-ref-1)
